# Graphene oxide in zinc alginate films: Antibacterial activity, cytotoxicity, zinc release, water sorption/diffusion, wettability and opacity

**DOI:** 10.1371/journal.pone.0212819

**Published:** 2019-03-07

**Authors:** Belén Frígols, Miguel Martí, Beatriz Salesa, Carolina Hernández-Oliver, Olav Aarstad, Ann-Sissel Teialeret Ulset, Gerd Inger Sӕtrom, Finn Lillelund Aachmann, Ángel Serrano-Aroca

**Affiliations:** 1 Facultad de Veterinaria y Ciencias Experimentales, Universidad Católica de Valencia San Vicente Mártir, Valencia, Spain; 2 NOBIPOL, Department of Biotechnology and Food Science NTNU Norwegian University of Science and Technology, Trondheim, Norway; Max-Planck-Institut fur Polymerforschung, GERMANY

## Abstract

Alginate is considered an exceptional biomaterial due to its hydrophilicity, biocompatibility, biodegradability, nontoxicity and low-cost in comparison with other biopolymers. We have recently demonstrated that the incorporation of 1% graphene oxide (GO) into alginate films crosslinked with Ca^2+^ cations provides antibacterial activity against *Staphylococcus aureus* and methicillin-resistant *Staphylococcus epidermidis*, and no cytotoxicity for human keratinocyte HaCaT cells. However, many other reports in literature have shown controversial results about the toxicity of GO demanding further investigation. Furthermore, the synergic effect of GO with other divalent cations with intrinsic antibacterial and cytotoxic activity such as Zn^2+^ has not been explored yet. Thus, here, two commercially available sodium alginates were characterised and utilized in the synthesis of zinc alginate films with GO following the same chemical route reported for the calcium alginate/GO composites. The results of this study showed that zinc release, water sorption/diffusion and wettability depended significantly on the type of alginate utilized. Furthermore, Zn^2+^ and GO produced alginate films with increased water diffusion, wettability and opacity. However, neither the combination of GO with Zn^2+^ nor the use of different types of sodium alginates modified the antibacterial activity and cytotoxicity of the zinc alginates against these Gram-positive pathogens and human cells respectively.

## Introduction

Alginate is considered a very promising material with exceptional hydrophilicity, biocompatibility, biodegradability, nontoxicity and low-cost in comparison with other biopolymers [1]. Its linear structure of (1–4)-linked β-d-mannuronic acid (M) and α-l-guluronic acid (G) residues is arranged in a block wise fashion with M and G present in different proportions and sequences depending on the source of alginate [2,3]. Divalent metallic cations such as Ca^2+^ and Zn^2+^ can interact with G-blocks to form, respectively, calcium and zinc alginate hydrogels arranged according to the classical *egg-box model* [4]. Calcium alginate exhibits negligible antibacterial and cytotoxic activity[5]. However, zinc alginates have shown strong antibacterial activity against a wide range of microorganisms [6–9] due to its inhibition of conserved metabolic pathways involved in synthesis of essential biomolecules or antioxidant depletion[10], and it is well-known that Zn^2+^ may produce toxic effects in human cells [11]. Alternative materials such as graphene oxide (GO), which from the graphene family shows the easiest processing, larger scale production and less expensive cost [12], has recently exhibited antibacterial capacity and negligible mammalian cytotoxicity in calcium alginate [5]. Thus, some researchers have paid particular attention to the incorporation of GO into polymers as promising candidates for the next generation of antibacterial agents [13–16]. Furthermore, nanocomposites such as polyvinyl-N-carbazole (PVK)-graphene oxide (GO) showed higher antimicrobial activity than pristine GO and no significant cytotoxicity to fibroblast cells [13]. The antimicrobial action of GO is usually associated with membrane disruption, bacteria wrapping, electron transfer mechanism and induction of oxidative stress by reactive oxygen species (ROS)[14,17]. However, the antibacterial activity and cytotoxicity of GO is still an opened question which demands further investigation because there are many controversial results under different conditions in literature. Thus, GO dispersions in isotonic saline solution in direct contact with bacterial cells showed strong antibacterial activity[18]. However, other studies with GO dispersions have shown no antibacterial activity [19] or even faster growth due to bacterial growth stimulation by GO surface for attachment and proliferation[20]. Furthermore, there are many studies that have stated that this carbon nanomaterial is toxic for some human cells[21,22].

On the other hand, GO is transparent in the visible spectrum [23] and is able to enhance many polymer properties such as water diffusion and mechanical performance [24–26] in a more efficient way than other carbon nanomaterials[27]. However, the transparency of GO decreases with increasing the presence of number of GO layers [28] ant thus, the darkness of polymer films can increase with increasing GO content [29]. Material wettability is another important surface property to be considered in polymer composite science for many applications industrial applications [30].

It is well known that the mechanical properties of alginate depend on chemical composition, sequence and molecular weight of the sodium alginate utilized [31]. Here, we hypothesize that some of other properties such as zinc release, water sorption/diffusion, wettability and/or opacity might also be affected by the type of commercial sodium alginate utilised and/or the incorporation of GO in the synthesis of zinc alginate films. However, as far as we know, hybrid materials such as GO in combination with Zn^2+^ in alginate films has never studied before. Thus, we assume increase of antibacterial activity upon the synergic effect of these two materials as a result of the intrinsic antibacterial activity of Zn [10] and that often reported for GO. Since GO nanosheets seems to be more effective against Gram-positive bacteria than Gram-negative bacteria [32,33], and we have shown that the inclusion of 1% *w/w* of GO into calcium alginate produced high growth inhibition on two clinically relevant Gram-positive bacteria (*Staphylococcus aureus* and methicillin-resistant *Staphylococcus epidermidis* (MRSE))[5], these two pathogens were tested on zinc alginates with and without the same amount of GO. *S*. *aureus* and *S*. *epidermidis* are currently considered two of the most important pathogens in nosocomial infections associated with catheters as well as other medical devices due to their natural abundance on skin [34]. MRSE strains are an important cause of catheter-associated disease, particularly among low birth weight premature infants [35].

Finally, since we have recently reported that the incorporation of 1% *w*/*w* of GO in calcium alginate produced reinforced films with no cytotoxicity for human keratinocyte HaCaT cells [5] and alginate is a biomaterial approved by the US Food and Drug Administration (FDA) for human wound healing [36], our third hypothesis stated that neither the addition of GO nor the use of different types of sodium alginates would modified the cytotoxicity of the zinc alginates.

## Materials and methods

### Materials

Sodium alginates were purchased and used as received from two commercial manufactures: Sigma-Aldrich (St. Louis, MO, USA) and Panreac AppliChem (Darmstadt, Germany), hereafter referred to as SA1 and SA2 respectively. Zinc chloride (≥ 97.0%, bioreagent for molecular biology suitable for cell culture), calcium chloride (≥ 93.0%) and graphene oxide were acquired from Sigma-Aldrich and used without further purification. Guluronate and mannuronate carbohydrate standards were provided by CarboSynth (Compton, Berkshire, UK). Glucose, trifluoro acetic acid, triethylenetetraminehexaacetic acid (TTHA), deuterium oxide (D_2_O, D-99.9%) and 3-(Trimethylsilyl)-propionic-2,2,3,3-d4 acid sodium salt (TSP) were supplied also from Sigma-Aldrich.

### Synthesis

Zinc alginate-based samples were synthesized following a chemical route recently reported for calcium alginate-GO composite films with enhanced physical properties in comparison to the conventional synthetic method of calcium alginates *via* bulk chelation with CaCl_2_ [37]. Thus, four type of zinc alginate samples were synthesized using SA1 and SA2 with and without 1% *w/w* of GO (referred to mass of sodium alginate), hereafter referred to as A1, A1GO, A2 and A2GO respectively. An amount of 0.5 grams of SA was dissolved in 44 mL of distilled water under continuous stirring for 1 hour (solution 1). After that, a solution 2 was prepared with 0.00505 grams of GO dispersed in 22 mL of distilled water. Subsequently, solution 1 and 2 were magnetically stirred for 10 minutes. The crosslinking solution was prepared dissolving 0.03 grams of zinc chloride in 10 mL of distilled water. This ZnCl_2_ aqueous solution was mixed with the GO/SA aqueous solution under magnetic stirring for 1 hour. The final mixture was cast to a glass Petri dish at 24±1°C for 24 hours to allow formation of thin film. Finally, the films were soaked in 2% (*w/v*) aqueous ZnCl_2_ solution for 2 hours, rinsed three times with distilled water, and vacuum dried at 60±0.5°C for 24 hours. The zinc alginate samples without GO were performed following the same procedure but without preparing solution 2. The amount of zinc in the samples was determined gravimetrically during the preparation procedure and found to be ∼12% *w/w*.

Control samples of calcium alginate, hereafter referred to as C1 and C2, were performed following the same procedure with SA1 and SA2, respectively, but without preparing solution 2 and using calcium chloride instead of zinc chloride as crosslinker agent.

### Characterization of alginates

Size exclusion chromatography with multi angel light scattering (SEC-MALS) detection was performed to determine molar mass and mass distribution of the two commercial sodium alginates (SA1 and SA2). Block length distribution of these sodium alginates was analysed by high performance anion-exchange chromatography with pulsed amperiometric detection (HPAEC-PAD). Finally, nuclear magnetic resonance (NMR) spectroscopy was performed to determine their fractions of M and G blocks (mono, di and triades).

SEC-MALS was performed at ambient temperature on a high-performance liquid chromatography (HPLC) system consisting of a solvent reservoir, online degasser, HPLC isocratic pump, automatic sample injector, pre-column, and serially connected columns (TSK 6000 and 5000 PW_XL_). The column outlet was connected to a Dawn HELEOS-II multiangle laser light scattering detector (Wyatt, U.S.A.) (λ_0_ = 663.8 nm) followed by Optilab T-rEX differential refractometer. The eluent was 0.15 M NaNO_3_/0.01 M EDTA (pH = 6) and the flow rate was 0.5 mL/min. Samples were filtered (pore size 0.8 μm) before injection. The injection volume was 100–500 μL, and the sample concentration was adjusted to obtain the best possible light scattering signal without influencing the refraction index (RI) profile (overloading). Data were collected and processed using the Astra (v. 6.1) software (Wyatt, U.S.A.). Three parallel injections of each sample were analysed.

SA1 (5.7 mg) and SA2 (8.8 mg) samples were suspended in 2M trifluoro acetic acid (TFA) to a final concentration of 2 g/L and further hydrolysed at 100°C for 12 hours. Thus, 1mL of each hydrolyte was dried on a centrifugal evaporator and then dissolved in Millipore-quality (MQ) water to a final concentration of 0.02 g/L. The samples were analysed by HPAEC-PAD on an ICS 5000+ system equipped with a pulsed amperometric detector (waveform A) using a CarboPac PA1 (4 x 50 mm) guard column and a 4x250 mm main column. The samples were eluted at a flow rate of 1 mL/min using the conditions shown in Table 1.

**Table 1:** HPAEC-PAD analysis conditions. Flow rates used in the HPAEC-PAD runs.

Time (min)	NaOH (mM)	NaAc (mM)	Gradient
0–25	10	0	isocratic
25–30	10–100	0	linear
30–40	100	0–200	linear
40–55	100	200	isocratic
55–60	100	0	isocratic
60–75	10	0	isocratic

The data obtained were processed using Chromeleon 7 Chromatography Data System software, version 7.2.1 and the peaks were identified using monosaccharide standards. The G block distribution were determined on HPAEC-PAD as previously described [38].

Briefly, the samples (1mg/mL) in 200mM Ammonium acetate, pH 7.2 with 50mM NaCl were added *Haliotis tuberculata* M-lyase (0.016 U/mg substrate) and incubated for 24 hours at 30°C. The lysates were analysed on an ICS 5000+ system using a 4x250 mm IonPac AS4A column with a 4x 50 mm AG4A guard column and a flow rate of 1mL/min. Samples were eluted with 100 mM NaOH and a linear sodium acetate gradient from 10–700 mM in 80 minutes.

NMR characterization was performed according to the ASTM F2259-10(2012)e1 for determining the chemical composition and sequence in alginate [39]. Thus, the samples were hydrolysed to an average chain length of 30–50 sugar units and freeze dried. Lyophilized samples (8–10 mg) were dissolved in D_2_O to a final volume of 600 μL D_2_O. TSP in D_2_O (2%, 5 μL) was added as the internal standard for the chemical shift. TTHA (0.3 M in D_2_O, pH 5.5) was added as a chelator to bind traces of Ca^2+^. ^1^H-NMR spectra were recorded at 84°C, which lies in the temperature range (80–90°C) usually utilised for alginates [40], on Bruker AVIIIHD 400 MHz equipped with 5mm SmartProbe 1D ^1^H spectra. Spectra were recorded using TopSpin 3.2 or 3.5 software (Bruker BioSpin) and processed and analysed with TopSpin 3.5 software (Bruker BioSpin). The viscosity of the high molecular weight samples was reduced by two-step acid hydrolysis to a final average DP_n_ around 30–50 according to the ASTM norm [39].

### Electron microscopy

The morphology of the GO powder utilized in this study was observed at a magnification of 50000 times by Field Emission Scanning Electron Microscopy (FESEM) with an Ultra 55 Model (Carl Zeiss, Switzerland) apparatus. High-resolution transmission electron microscopy (HR-TEM) with a JEM 2100F JEOL 200 kV electron microscope provided with Energy-Disperse X-Ray Spectroscopy operating at 20 kV for elemental analysis was performed. HR-TEM sample preparation consisted of dispersing GO in dichloromethane in an ultrasonic bath for 10 minutes and subsequent drying of one drop on a grid at ambient temperature for 5 minutes prior to observation.

A JEM-1010 (JEOL, Japan) 100 kV transmission electron microscope (TEM) was utilised to observe the nanostructure of the zinc alginate/GO composite films. Ultrathin sample preparation with sections of 60 nm was performed with a Leica Ultracut UC6 ultramicrotome (Leica Mikrosysteme GmbH, Austria) and a Diatome diamond knife (Diatome Ltd., Switzerland). The specimens were placed on TEM grids (300 mesh) coated in carbon film.

### Zinc release, antibacterial activity and cytotoxicity

Due to these hydrophilic materials are brittle in the dry state, the films were hydrated in distilled water for 1 hour to be able to be cut into disk shapes of 10 mm diameter. Then, the sample disks were dried at 60°C for 24 hours. Each specimen was sterilized with ethanol and ultraviolet (UV) radiation for 1 hour per side inside a laminar flow hood with a 12.0 W lamp of UV-C radiation right before antibacterial and cytotoxic characterization.

In order to quantify amount of zinc released from films, the sample disks were incubated individually in a 48-well plate with 600 μL of MQ water at 37°C. Every 24 hours, the 600 μL of MQ water were collected and replaced during 5 days and finally at day 10. The zinc measurements were performed using the Zincon Assay (Sigma-Aldrich, St Louis, MO, USA). Absorbance at 570 nm was read on a microplate reader (Varioskan, Thermo Fisher).

The antibacterial activity of the produced materials was tested on Gram-positive bacterium *Staphylococcus aureus*, V329 [41] and *Staphylococcus epidermidis*, RP62A [42], an MRSE strain by the agar disk diffusion test [43,44].

Lawns of test bacteria of about 1.5×10^8^ colony forming units per millilitre (CFU/mL) were prepared on tryptic soy agar (TSA) from Liofilchem (Teramo, Italy). The sterilized samples were carefully placed upon the lawns and incubated at 37°C for 24 hours. The inhibitory action of tested samples on the growth of the bacteria was determined by measuring diameter of inhibition zone (*d*_*iz*_) and sample disk diameter (*d)* with an image analysis software (Image J). Then, the normalised width of the antimicrobial “halo” (*nw*_*halo*_) of each sample was calculated by applying Eq (1) [43].

$$nw_{halo}=\frac{\frac{d_{iz}-d}{2}}{d}$$ (1)

Each antibacterial test was performed in quadruplicate in four different days to ensure reproducibility. Thus, the mean and standard deviation of the normalised antibacterial “halos” were determined.

The ISO-10993 standard recommendations were followed in these cytotoxicity assays. Thus, the sterilised sample disks (n = 4) were placed into a well of a 12-well plate with 1 mL of DMEM (Biowest SAS, France) without fetal bovine serum (FBS, Biowest), covering the whole surface area. A volume ratio of 3 cm^2^/mL was selected according to this norm. After being incubated the sample disks in humidified 5% CO_2_/95% air atmosphere for 72 hours at 37°C, the extracts were collected and filtered with a 0.20 μm filter unit, and used immediately for the cytotoxic assay.

Non-tumorigenic immortalized human keratinocyte HaCaT cells were obtained from the Medical Research Institute Hospital La Fe, Spain. These cells were cultured in DMEM containing 10% FBS, 100 units/mL penicillin (Lonza, Belgium) and 100 mg/mL streptomycin (HyClone, GE Healthcare Life Sciences) in a 37°C, 5% CO2 incubator.

The effect of the treatment with the sample disk extracts on cell viability was measured by the methylthiazolyldiphenyl-tetrazolium bromide (MTT) assay. Thus, cells were planted onto 96-well plate at a density of 5 × 10^5^ cells/well. After incubation for 24 hours at 37°C in a 5% CO_2_ humidified atmosphere, the 100 μL of medium in each well was replaced with 100 μL of undiluted extract or control medium without FBS (the same as used to produce the extracts). Diluted extracts at a concentration of 10, 5 and 1% *v/v* were also tested with samples A1, A1GO, A2 and A2GO. The cells in each well were incubated with 5 mg/mL MTT for 4 h. The formazan was dissolved in 100 μL dimethyl sulfoxide (DMSO, Sigma-Aldrich) at room temperature. Absorbance at 490 nm was read on a microplate reader (Varioskan, Thermo Fisher).

### Water sorption and diffusion studies

Water contents (*w/w*), defined as grams of absorbed water per gram of dry polymer, were measured by immersion experiments at body temperature (37±0.5°C). Samples with approximate dimensions of 3 × 3 cm were located in a thermostatic water bath and weighed at specific time intervals after removing the water remaining on their surface with absorbent paper. In this kind of experiments, water content increases to reach an equilibrium water content (*w*_*eq*_) providing useful information related to water sorption and diffusion. These measurements were conducted in quadruplicate to confirm the reproducibility of the results.

From these measurements of liquid water sorption, an apparent diffusion coefficient (*D*) can be determined to compare the diffusion process in all these systems, assuming that the immersion of these hydrophilic materials in water obey Fick’s law. Thus, a linear regression with Eq (2) can be performed representing *Δm*_*1*,*t*_/*Δm*_*1*,*∞*_ vs. *t*^*1/2*^*/l* for relatively small values of time *t* or *Δm*_*1*,*t*_/*Δm*_*1*,*∞*_ < 0.5 [45].

$$\frac{\Delta m_{1,t}}{\Delta m_{1,\infty }}\approx 4{\left(\frac{Dt}{\pi \cdot l^{2}}\right)}^{\frac{1}{2}}$$ (2)

where *Δm*_*1*,*t*_ and *Δm*_*1*,*∞*_ are the weight increments at time *t* and at equilibrium, respectively and *l* is the sample thickness.

### Wettability measurements

Contact angle and Surface Free Energy (SFE) were determined on rectangular bar specimens with approximate dimensions of 1 × 4 cm by the sessile drop method. Ten 5 μL droplets of two liquids with opposite polarity and known surface tension were used [46]: water (Y_1_ = 72.8 mN/m^2^) and methylene iodide (Y_1_ = 50.8 mN/m^2^). Y_1_ refers to the total surface free energy of the liquid. The contact angle was then measured after placing the drop on the material surface by imaging the drop with a high-resolution digital camera as soon as possible at 24±1°C. The profile of the drop was then processed with ImageJ software to determine the contact angles.

The surface free energy (*Y*_*s*_) of these materials was determined by the Fowkes’[47] and Owens’[46] method applying the following equations:

$$Y_{s}=Y_{s}^{d}+Y_{s}^{p}$$ (3)

$$\left(1+cos\theta \right)Y_{l}/2={\left(Y_{s}^{d}Y_{l}^{d}\right)}^{1/2}+{\left(Y_{s}^{p}Y_{l}^{p}\right)}^{1/2}$$ (4)

where $Y_{s}^{d}$, $Y_{s}^{p}$, $Y_{l}^{d}$ and $Y_{l}^{p}$ are the dispersive (d) and polar (p) components of the solid (s) and the liquid (l), respectively. The polar and dispersive values for the tested liquids (water and methylene iodide) were taken from the literature[48].

### Opacity

The opacity of the films was determined according to the following Eq [49]:

$$\text{O}=\frac{\text{Abs}600}{\text{x}}$$ (5)

where Abs600 is the value of absorbance at 600 nm and x is film thickness (mm).

Thus, rectangular sample films with approximate dimensions of 4 mm×50 mm, and vacuum dried at 60°C for 24 hours, were directly placed in a spectrophotometer cell to measure the film absorbance at 600 nm with a UV/VIS spectrophotometer (Nanocolor UV0245, Macherey-Nagel, Germany). An empty test cell was used as reference in these measurements. Results were reported as absorbance divided by film thickness (mm) based on four replications to confirm reproducibility.

### Statistical analysis

The results obtained in this study were analysed by ANOVA, followed by Turkey’s post hoc analysis with SPSS22 at significance level of p < 0.05.

## Results and discussion

### Characterization of alginates

The HPAEC-PAD monosaccharide analysis of the two commercial sodium alginates showed that one of them is pure alginate (SA1) while the other contains 14% α-d-glucose (SA2). Furthermore, the two alginates differ in chain length (3x) and composition. Thus, Table 2 shows number average molecular mass (M_n_), average molecular mass (M_w_) and polydispersity of the Sigma-Aldrich alginate (SA1) and the AppliChem alginate (SA2).

**Table 2:** Molecular mass and polydispersity of the analysed sodium alginates. Number average molecular mass (M_n_), average molecular mass (M_w_) and polydispersity of the Sigma-Aldrich alginate (SA1) and the AppliChem alginate (SA2).

Sample	M_n_ (kDa)	M_w_ (kDa)	Polydispersity (M_w_/M_n_)
SA1	60.5±5.8	107.9±2.7	1.79±0.13
SA2	170.7±3.1	379.5±9.5	2.22±0.08

Analyses by NMR reveals the content of the two monomers of alginate, as well as the distribution through the polymer (see Table 3). The M/G ratio is quite similar for both alginates with F(G) around 0.43 and the G-block length is similar for both alginates.

**Table 3:** Guluronic/Mannuronic ratios of the analysed sodium alginates. Distribution of Guluronic (G) and Mannuronic (M) acid content for the two analysed SA1 and SA2 alginates.

Blocks	SA1	SA2
F(G)	0.436	0.427
F(M)	0.564	0.573
F(GG)	0.251	0.270
F(GGG)	0.202	0.234

**Fig 1:**
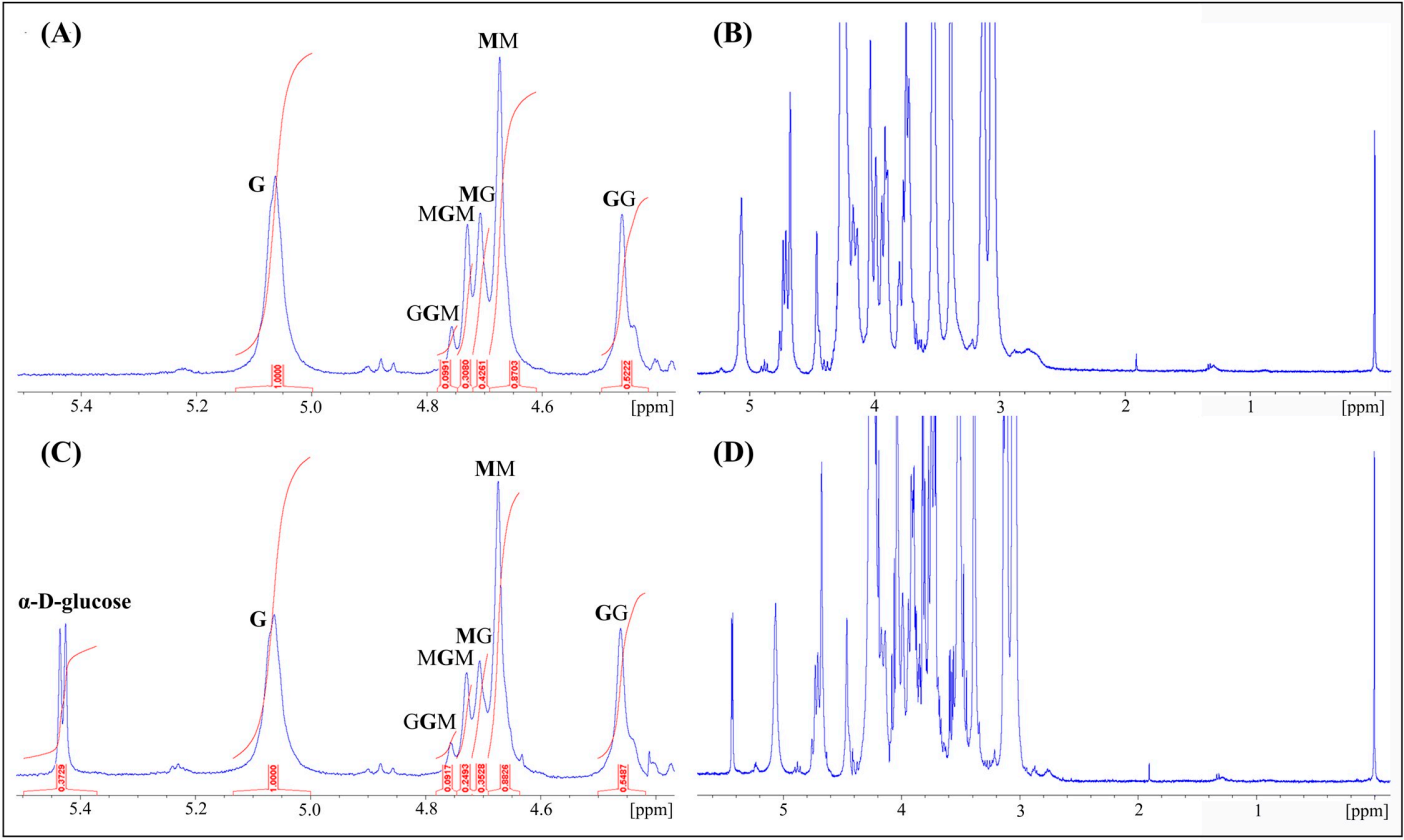
^1^H-NMR analysis of the sodium alginates. ^1^H-NMR spectra recorded at 400 MHz and 84°C. (A) Anomeric region of partly hydrolysed Sigma-Aldrich (SA1) alginate. (B) Full ^1^H-NMR spectra of partly hydrolysed SA1 alginate. (C) Anomeric region of the ^1^HNMR spectra of partly hydrolysed Applichem (SA2) alginate. (D) Full ^1^H-NMR spectra of partly hydrolysed SA2 alginate.

Despite the similarities, a noticeable difference can be seen in form of a doublet at 5.32 ppm (^3^J coupling of 5.3 Hz) in the spectra of the SA2 alginate (Fig 1). This indicate that the SA2 sample contains α-d-glucose (starch).

More detailed information about the SEC-MALS and HPAEC-PAD characterization of the two commercially available alginates is available in the Supporting information.

### Electron microscopy

**Fig 2:**
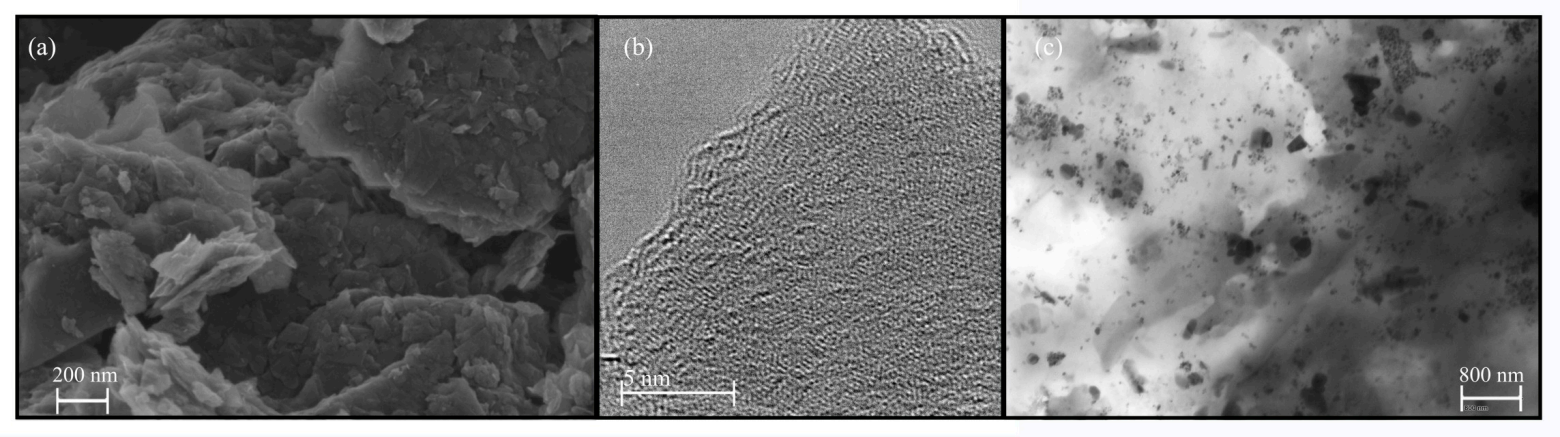
Electron microscopy. Electron microscopy images: (a) FESEM and (b) HR-TEM of graphene oxide powder; (c) TEM morphology of calcium alginate/graphene oxide film (sample A2GO).

The GO powder utilized in the synthesis of the GO/zinc alginate composite films (A1GO and A2GO) present a morphology of irregular nanosheets forming aggregates due to van der Waals forces and π-π interactions (see Fig 2A).

Fig 2B shows at higher magnification the atomic carbon structure of a single GO nanosheet by HR-TEM, in which the GO elementary analysis showed a composition of 98.5 and 1.5% *w/w* for C and O elements respectively.

Fig 2C shows the nanostructure of a zinc alginate composite film with GO (sample A2GO) composed of alginate (light phase) and GO (dark phase) embedded in the alginate polymer matrix and randomly distributed. It is of note that the GO nanosheets tend to aggregate forming GO networks crosslinked with divalent cations by coordination chemistry as already reported [24,50]. The TEM morphology of this sample is not very different from that of the other composite film synthesised with GO and SA1 (image not shown). It is important to mention that the composite films were produced with an enhanced chemical route that yields nanocomposites with enhanced tensile modulus, wettability, water diffusion and thermal properties[37]

### Zinc release

**Fig 3:**
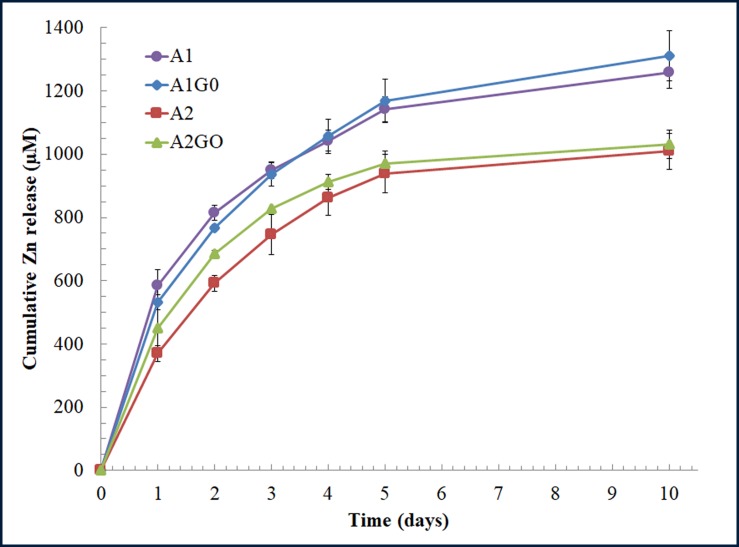
Zn release analysis. Zn release (μM) from the zinc alginates with (A1GO and A2GO) and without (A1and A2) 1% *w/w* of graphene oxide after immersion in MQ water at 37°C during 10 days.

Zinc alginate films were prepared using the two characterized sodium alginates with and without 1% *w/w* of GO. Thus, Fig 3 shows the cumulative Zn release profiles of the samples are over the reported zinc cytotoxicity level of 100 μM reported for mesenchymal stromal cells [51].

Within the experimental uncertainty, the Zn release from all the samples is quite similar after 1 day of immersion in MQ water at 37°C. However, after 10 days, the samples prepared with SA1 (samples A1 and A1GO) showed statistically significant higher cumulative Zn release than those prepared with SA2. Since high mannuronic (M) acid alginates bind divalent cations less strongly than high guluronic (G) acid alginates [52] and both SA1 and SA2 sodium alginates present similar M/G ratio (see Table 3), these differences of zinc release can be attributed to the fact that SA1 have three times lower molecular mass than SA2 (see Table 2). Similar zinc release dependence on molecular weight has been reported for other polymers such as PLGA or PLA [53]. However, the incorporation of GO into both type of zinc alginates did not show any statistically significant effect on Zn release.

### Antibacterial activity

**Fig 4:**
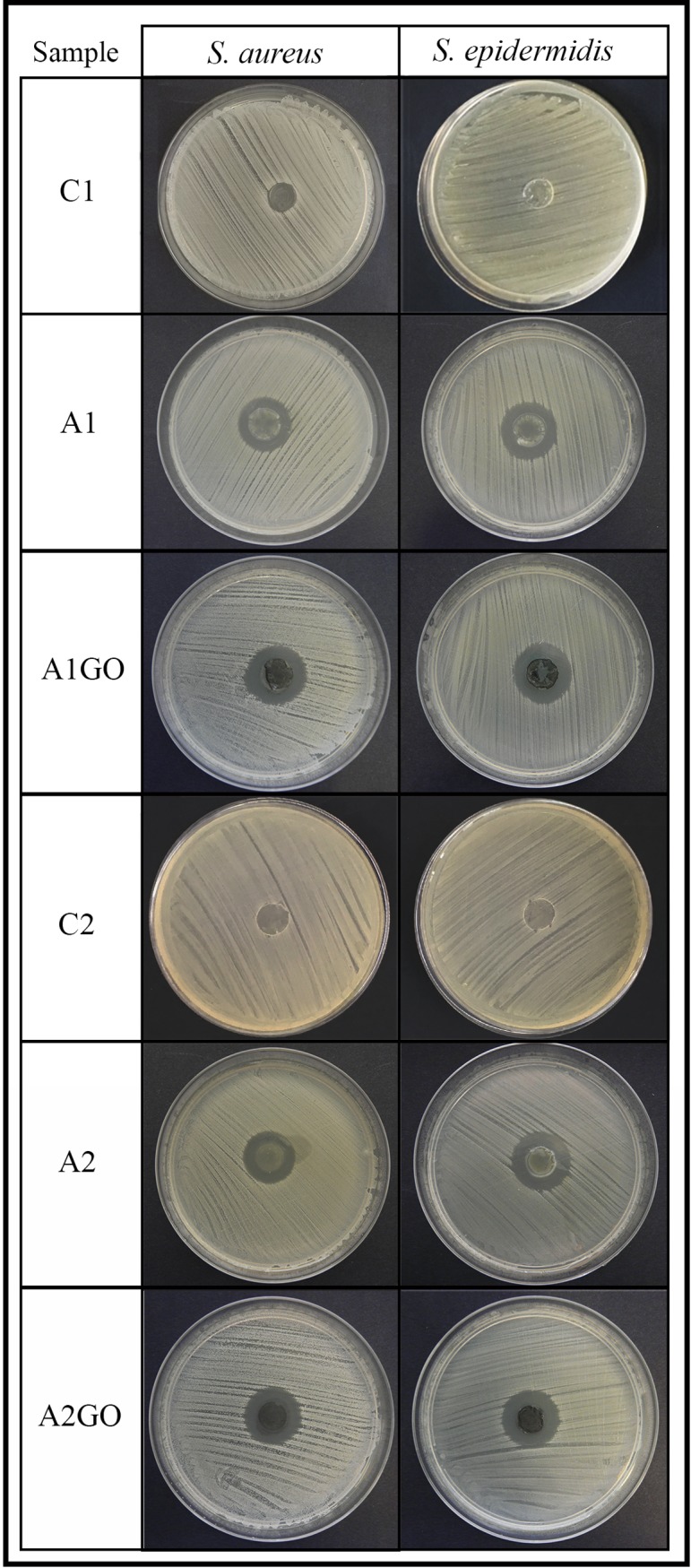
Antibacterial tests by the agar disk diffusion test. Antibacterial activity of the zinc alginates with (A1GO and A2GO) and without (A1and A2) 1% *w/w* of graphene oxide against *Staphylococcus aureus* and methicillin-resistant *Staphylococcus epidermidis* after 24 hours of culture. Negative results of the calcium alginate control samples are referred to as C1 and C2.

The agar disk diffusion test showed that all zinc alginates with (A1GO and A2GO) and without GO (A1 and A2) possessed strong antibacterial activity against the two tested Gram-positive strains (see Fig 4). The calcium alginate films (C1 and C2) prepared with the two sodium alginates did not showed any inhibition zone as expected [54,55].

The normalised width of the antimicrobial “halo” (*nw*_*halo*_) of each sample was calculated using Eq (1) and showed neither statistically significant increase of antibacterial activity by the incorporation of GO into the zinc alginates nor by the use of different commercial sodium alginates against both strains (results shown in S1 Table in the Supporting information). These similar results of *nw*_*halo*_ (~0.48) after one day of bacterial culture could be attributed to the fact that the four samples release zinc at a similar speed during the first day (see Fig 3). However, it is of note that these antibacterial films with and without GO possess much higher antibacterial capacity than calcium alginate/GO films prepared with the same GO content (*nw*_*halo*_ ~0.27) against *S*. *aureus* and MRSE respectively [5].

### Cytotoxicity assay with extracts

**Fig 5:**
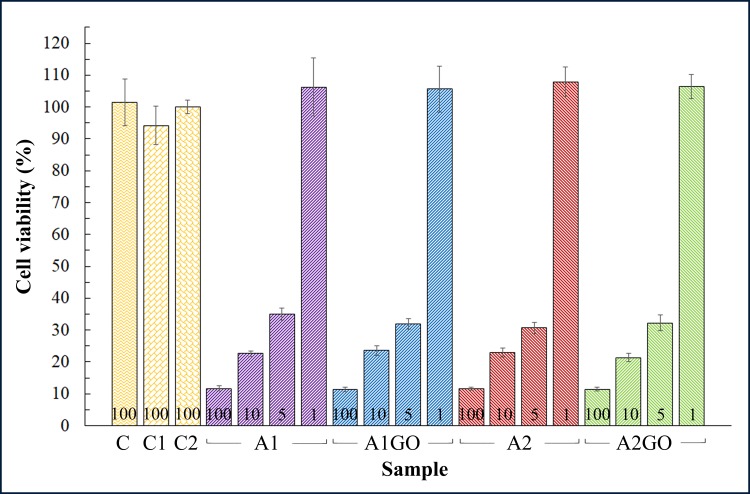
Cytotoxicity analysis. Cell viability (%) of extracts of zinc alginate films with (A1GO and A2GO) and without (A1 and A2) graphene oxide, control culture medium (C) and extracts of the negative control calcium alginate films (C1 and C2) in the presence of human keratinocyte HaCaT cells. Numbers 100, 10, 5 and 1 depict the concentration (% *v/v*) of the extracts. Neither statistically significant differences (p<0.05) of cell viability were found between the extracts of A1, A1GO, A2 and A2GO samples with similar concentrations, nor between control extracts and sample extracts at 1% *v/v*.

The results of the cytotoxicity tests performed with the extracts in the presence of human keratinocyte HaCaT cells are shown in Fig 5.

Non-diluted extracts of all the samples containing zinc showed very strong cytotoxicity. This toxicity to HaCaT cells decreased more and more by diluting the extracts at lower and lower concentrations. Thus, the extracts diluted at a concentration of 1% *v/v*, which corresponds to a zinc concentration (see Fig 3) below the toxic level (~100 μM) for mesenchymal stromal cells [51], showed no cytotoxic activity as expected.

These results showed no statistically significant differences in cell viability (%) neither by the inclusion of GO nor the use of different sodium alginates in the tested samples.

### Water sorption and diffusion, wettability and opacity measurements

Equilibrium water contents (*w*_*eq*_) and apparent diffusion coefficients (*D*) determined with Eq (2) at 37°C for each sample are shown in Table 4. These results showed that the addition of 1% *w/w* of GO in both types of commercial sodium alginates produced a significant reduction of equilibrium water sorption and increase of water diffusion due to the formation of carbon nanochannels, which tighten the polymer network and through which water can penetrate faster [24].

**Table 4:** Water sorption and diffusion, wettability and opacity results. Water and methylene iodide contact angle (*θ*), solid surface free energy (*Y*_*s*_) with their components (dispersive $Y_{s}^{d}$ and polar $Y_{s}^{p}$), equilibrium water contents at 37°C (*w*_*eq*_), apparent diffusion coefficient (*D*) at 37°C (assuming Fick’s law is obeyed) and opacity (*O*) values of the zinc alginate films with (A1GO and A2GO) and without (A1and A2) 1% *w/w* of graphene oxide and control samples of calcium alginate (C1 and C2). Data are shown as mean±standard deviation. * Different superscript letters in values of the same column indicate statistically significant differences (p<0.05).

SAMPLE	*θ* (H_2_O)	*θ* (CH_2_I_2_)	$Y_{s}^{d}$(mN/m)	$Y_{s}^{p}$(mN/m)	*Y*_*s*_(mN/m)	*w*_*eq*_	*D*·10^7^ (cm^2^/s)	*O* (Abs_600_/mm)
C1	46.9±4.2	54.4±4.3	31.8±2.5	23.9±3.3	55.5±2.7^a^*	0.95±0.08^a^*	0.65±0.11^a^*	1.58±0.22 ^a^*
A1	48.1±2.7	53.5±3.1	32.9±1.8	22.6±2.2	55.2±1.7^a^*	0.97±0.11^a^	0.60±0.12^a^*	1.65±0.17^a^*
A1GO	42.0±3.1	49.3±3.1	35.3±1.8	25.0±2.3	60.0±1.8^b^*	0.71±0.09^b^	0.90±0.13^b^*	6.73±0.08^b^*
C2	67.5±3.8	46.8±5.2	36.7±2.8	13.2±4.7	46.1±2.3^c^*	0.92±0.12^a^	2.71±0.23^c^*	1.67±0.24^a^*
A2	65.8±2.5	49.4±2.1	35.3±1.2	10.9±1.2	45.8±1.7^c^*	0.96±0.09^a^	2.60±0.26^c^*	1.79±0.10^a^*
A2GO	58.5±2.3	47.0±2.6	36.6±1.5	14.5±1.7	50.7±1.2^d^*	0.74±0.08^b^	3.72±0.26^d^*	6.69±0.28^b^*

No statistically significant effect of the type of sodium alginate utilized was observed on water sorption equilibrium. However, a strong dependence of *D* on the commercial sodium alginate used in the synthesis was revealed in these experiments. Thus, SA2 significantly increased *D* in both calcium and zinc alginates. According to the alginate characterization results, these samples contain α-d-glucose, which like most plasticizers contain hydroxyl groups which enhance film flexibility [56]. Thus, it has been reported that the diffusion coefficient (*D*) across the alginate films increases with addition of plasticizers because the leaching of these compounds could reduce tortuosity of aqueous pore channels of the films [57]. This fact could explain why C2, A2 and A2GO synthesized with SA2 exhibited higher water diffusion coefficients than the samples synthesized with pure sodium alginate SA1 (C1, A1 and A1GO).

Wettability experiments showed that contact angle values (*θ*) significantly decreased in both alginates with the addition of GO, which resulted in a significant increase of surface free energy (*Y*_*s*_), that is, more hydrophilic as expected [58] (see Table 4). The films synthesized with SA1 exhibited higher hydrophilicity than those prepared with SA2 demonstrating the importance of alginate selection in the synthesis of alginate-based materials for certain applications where this property is relevant. This increase of water contact angle and thus decrease of surface tension of the samples prepared with SA2 with respect to those synthesized with SA1, could be also attributed to the amount glucose (14%) present in these samples as it occurred in a previous study with the addition of a polysaccharide in alginate [59].

Finally, opacity, which is an established measurement of the transparency of a film, showed in Table 4 no statistically significant differences (p<0.05) between alginate films (C1, A1, C2 and A2). However, the opacity of both types of zinc alginate increases dramatically by incorporating just 1% *w/w* of GO, which should be taking into account for certain biomedical fields demanding transparent materials such as ophthalmology and odontology. It is of notice that the opacity achieved with Zn^2+^ cations and GO in these alginates is almost double than that achieved with Ca^2+^ and the same amount of GO[5]. This difference of opacity can be attributed to the fact that Zn^2+^ is more effective crosslinker of GO [50] and thus can form more crosslinked GO networks inside the alginate matrix than Ca^2+^ [24], which darkens more the zinc alginate composites [50].

Therefore, zinc release, water sorption/diffusion, wettability and opacity are affected by the type of commercial sodium alginate utilised and/or the inclusion of GO in the synthesis of zinc alginate films in good agreement with our fist hypothesis. In addition, these results are also consistent with our third hypothesis of no cytotoxic modification produced by the use of different types of sodium alginates or the addition of GO into the zinc alginates. However, the incorporation of GO did not increase the antibacterial capacity of the different zinc alginates, contrary to what we expected.

## Conclusions

Two commercial sodium alginates were characterized and used to synthesize zinc alginate films with and without 1% *w/w* graphene oxide. These advanced materials exhibited very strong antibacterial activity against Gram-positive *Staphylococcus aureus* and methicillin-resistant *Staphylococcus epidermidis* only due to the presence of Zn^2+^. Several properties of these hydrophilic materials such as zinc release, water sorption/diffusion, wettability and opacity of these materials showed to be significantly affected by the type of sodium alginate utilised and/or the incorporation of 1% *w/w* of GO. However, neither the incorporation of GO nor the use of different types of sodium alginates increased the cytotoxicity of the films in human keratinocyte HaCaT cells.

## Supporting information

S1 Fig SEC-MALLS chromatograms. SEC-MALLS analysis of Sigma-Aldrich alginate (SA1) in red and AppliChem alginate (SA2) in black.
(TIF)

S2 Fig HPAEC-PAD monosaccharide analysis. Analysis of Sigma-Aldrich (SA1) and Applichem (SA2) samples. (A) Region where alditol and neutral monosaccharides elutes and (B) Acidic monosaccharides.
(TIF)

S3 Fig HPAEC-PAD chromatograms. Overlaid HPAEC-PAD chromatograms of: (A) G-block partially degraded with G-lyase compared with M-lyase degraded alginate from (B), *Laminaria hyperborea* stipe (67% G), (C) AppliChem (SA2) and (D) Sigma-Aldrich (SA1) samples. Some shorter oligomers are identified in the figure together with G-blocks up to DP 50. Unsaturated non-reducing ends are denoted by Δ.
(TIF)

S1 Table Antimicrobial results by the agar disk diffusion test. Antimicrobial “halo” (*nw*_*halo*_) of zinc alginate with (A1GO and A2GO) and without (A1 and A2) 1% *w/w* of graphene oxide by the agar disk diffusion test against the tested *S*. *aureus* and MRSE strains and calculated with Eq (1). The negative results obtained with the control calcium alginate samples (C1 and C2) are also indicated.
(DOCX)
