# Supplementary figures and images for: Graphene oxide in zinc alginate films: Antibacterial activity, cytotoxicity, zinc release, water sorption/diffusion, wettability and opacity
Source: PLoS One. 2019 Mar 7;14(3):e0212819. doi: 10.1371/journal.pone.0212819 (PMC6405205; doi:10.1371/journal.pone.0212819)

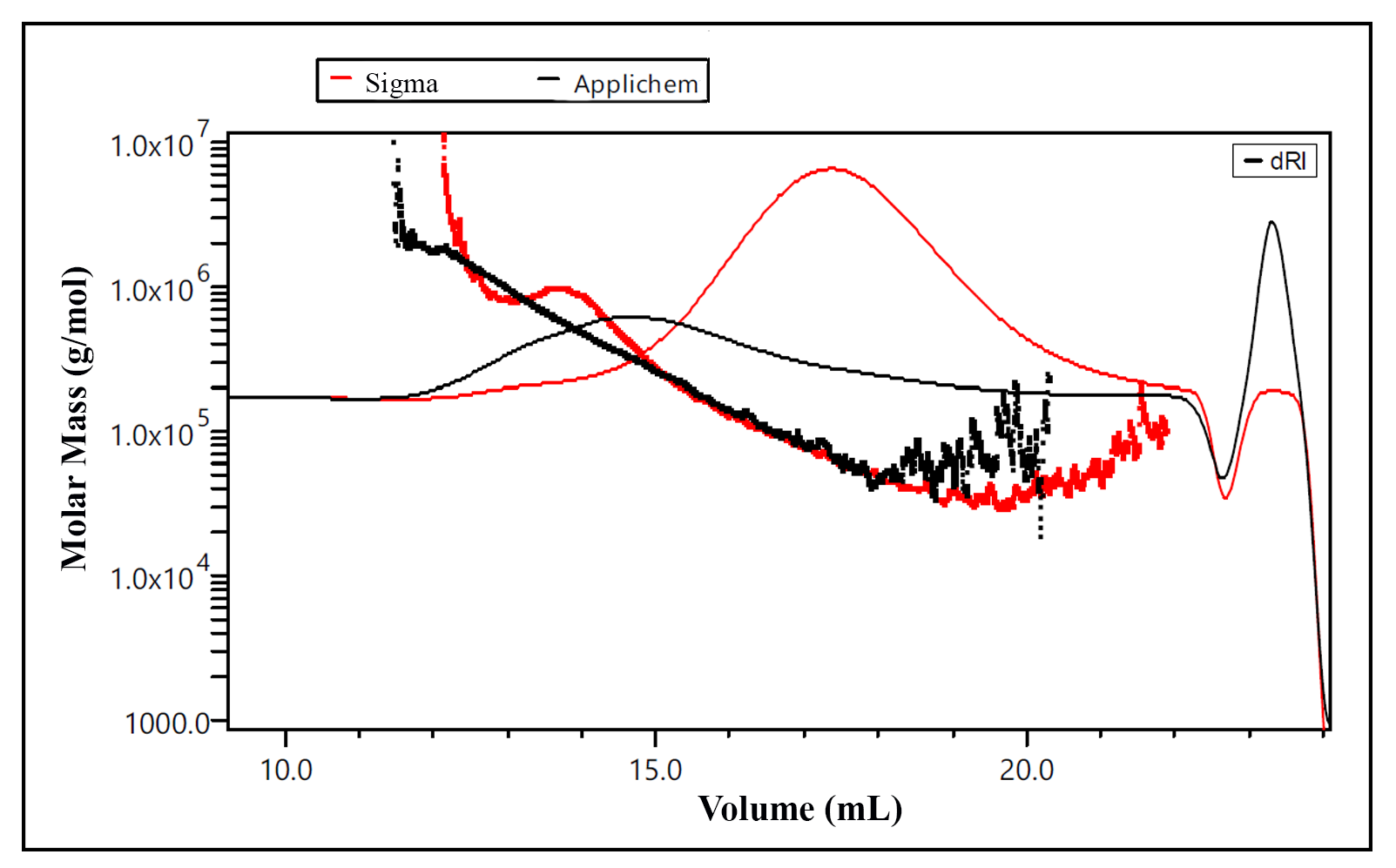

Supplement: S1 Fig — SEC-MALLS analysis of Sigma-Aldrich alginate (SA1) in red and AppliChem alginate (SA2) in black. (TIF) [file pone.0212819.s001.tif]

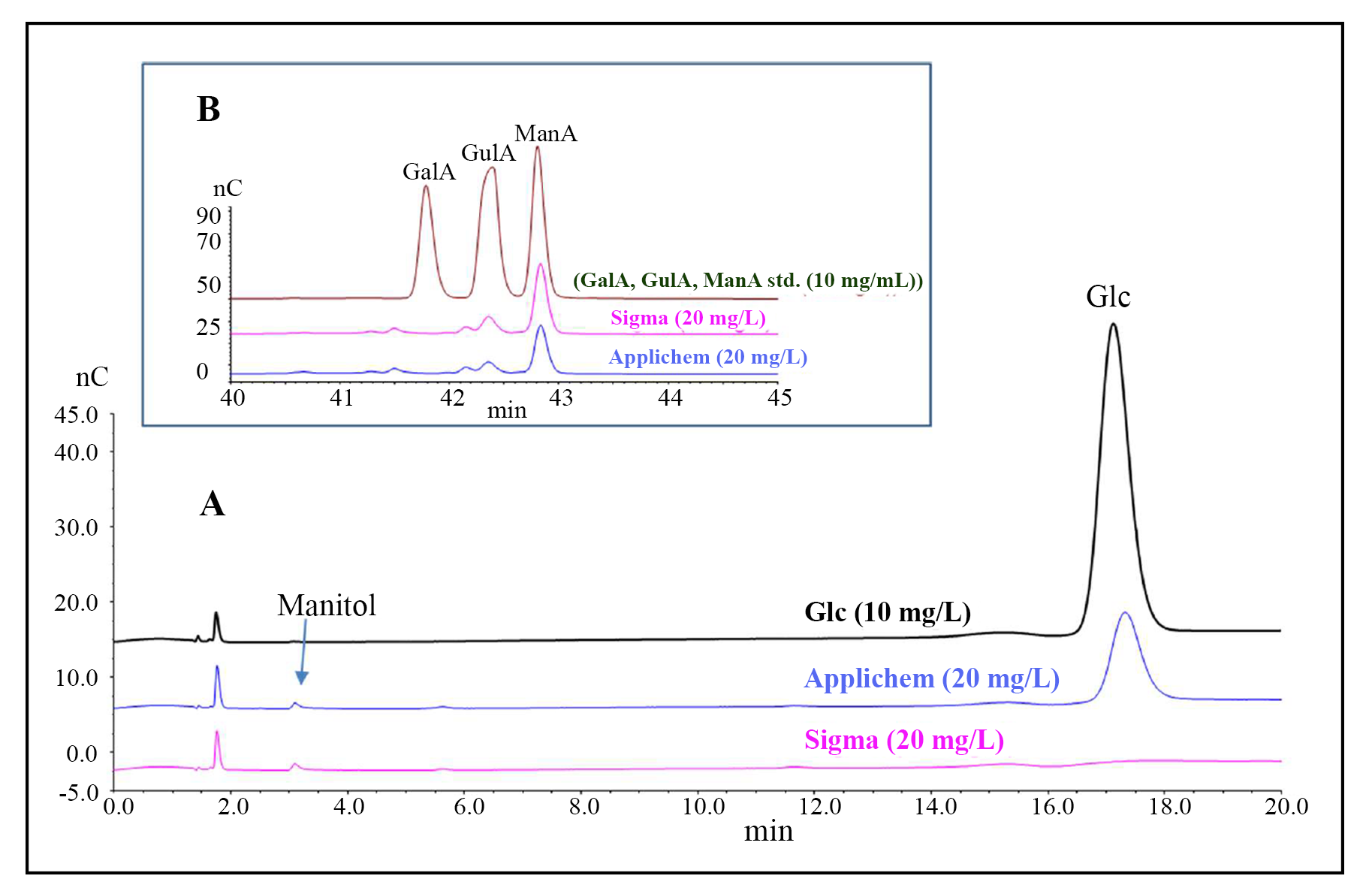

Supplement: S2 Fig — Analysis of Sigma-Aldrich (SA1) and Applichem (SA2) samples. (A) Region where alditol and neutral monosaccharides elutes and (B) Acidic monosaccharides. (TIF) [file pone.0212819.s002.tif]

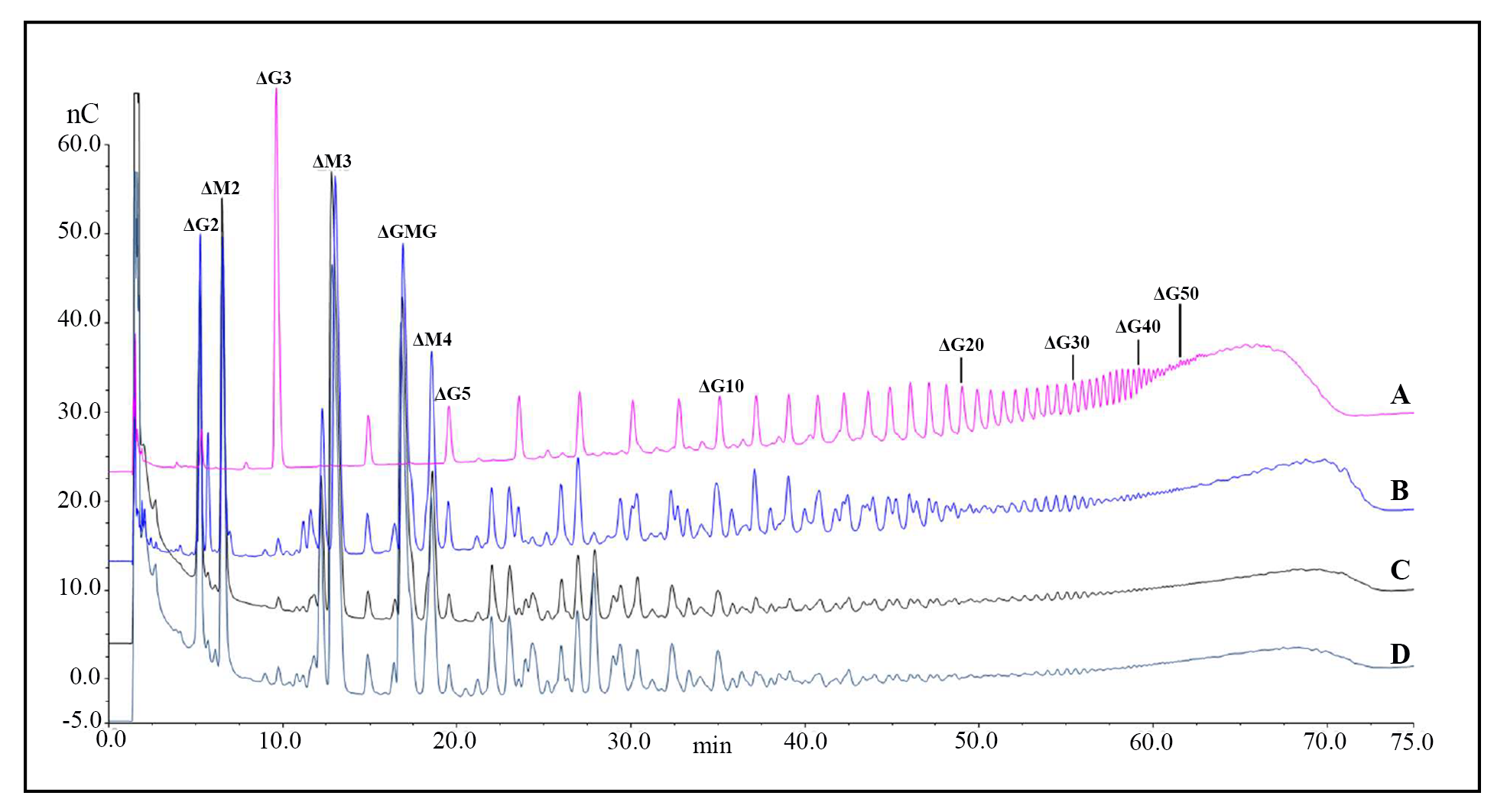

Supplement: S3 Fig — Overlaid HPAEC-PAD chromatograms of: (A) G-block partially degraded with G-lyase compared with M-lyase degraded alginate from (B), Laminaria hyperborea stipe (67% G), (C) AppliChem (SA2) and (D) Sigma-Aldrich (SA1) samples. Some shorter oligomers are identified in the figure together with G-blocks up to DP 50. Unsaturated non-reducing ends are denoted by Δ. (TIF) [file pone.0212819.s003.tif]
