# Supplementary material for: Graphene oxide in zinc alginate films: Antibacterial activity, cytotoxicity, zinc release, water sorption/diffusion, wettability and opacity
Source: PLoS One. 2019 Mar 7;14(3):e0212819. doi: 10.1371/journal.pone.0212819 (PMC6405205; doi:10.1371/journal.pone.0212819)
Supplement: S1 Table — Antimicrobial “halo” (nwhalo) of zinc alginate with (A1GO and A2GO) and without (A1 and A2) 1% w/w of graphene oxide by the agar disk diffusion test against the tested S. aureus and MRSE strains and calculated with Eq (1). The negative results obtained with the control calcium alginate samples (C1 and C2) are also indicated. (DOCX) [file pone.0212819.s004.docx]

**Supporting information**

## Graphene oxide in zinc alginate films: Antibacterial activity, cytotoxicity, zinc release, water sorption/diffusion, wettability and opacity

Belén Frígols^1¶^, Miguel Martí^1¶^, Beatriz Salesa^1¶^, Carolina Hernández-Oliver^1¶^, Olav Aarstad^2^, Ann-Sissel Teialeret Ulset^2^, Gerd Inger Sӕtrom^2^, Finn Lillelund Aachmann^2^, Ángel Serrano-Aroca^1*^

^1^ Facultad de Veterinaria y Ciencias Experimentales, Universidad Católica de Valencia San Vicente Mártir, Valencia, Spain.

^2^ NOBIPOL, Department of Biotechnology and Food Science NTNU Norwegian University of Science and Technology, Trondheim, Norway.

^¶^ These authors contributed equally to this work

^*^ Corresponding author

E-mail: [angel.serrano@ucv.es](mailto:angel.serrano@ucv.es) (AS)

**Supporting information**

**Characterization of alginates**

**Molecular weight and polydispersity**

The number average molecular mass (M_n_), average molecular mass (M_w_) and polydispersity (Table 2) and the SEC-MALLS chromatogram (S1 Fig) showed that the two analysed alginate samples differ, both in chain length and polydispersity.

**S1 Fig.** **SEC-MALLS chromatograms.** SEC-MALLS analysis of Sigma-Aldrich alginate (SA1) in red and AppliChem alginate (SA2) in black.

**Monosaccharide analysis**

It is well known that Guluronic and Mannuronic acid monomers are prone to degradation,

mainly by decarboxylation during acid hydrolysis[67]. Both total and relative yield is therefore not accurate. The main purpose of this analysis was to check the purity of the SA1 and SA2 samples with regard to other monosaccharides (see S2 Fig).

**S2 Fig.** **HPAEC-PAD monosaccharide analysis.** Analysis of Sigma-Aldrich (SA1) and Applichem (SA2) samples. (A) Region where alditol and neutral monosaccharides elutes and (B) Acidic monosaccharides.

Traces of mannitol were identified in both samples. Most strikingly, the SA2 sample contains a significant amount of α-D-Glucose (starch), calculated to constitute around 14% of the dry weight.

**Analysis of G block distribution in alginate**

The HPAEC-PAD chromatograms of the lyase degraded SA1 and SA2 samples together with standards are shown in S3 Fig.

**S3 Fig.** **HPAEC-PAD chromatograms.** Overlaid HPAEC-PAD chromatograms of: (A) G-block partially degraded with G-lyase compared with M-lyase degraded alginate from (B), *Laminaria hyperborea* stipe (67% G), (C) AppliChem (SA2) and (D) Sigma-Aldrich (SA1) samples. Some shorter oligomers are identified in the figure together with G-blocks up to DP 50. Unsaturated non-reducing ends are denoted by Δ.

The lyase degraded seaweeds contains a more complex mixture of oligomers than the G-block standard, with lack of resolution already from DP> 5. The reason is that the M- lyase is not able to cleave 1-2 M units on each side of the G block. Although HPAEC-PAD is in principle a quantitative technique, the lack of standards, together with lacking resolution for these complex samples leads to chromatograms that should be regarded more as fingerprints than a quantitative analysis. However, some main characteristics may still be extracted. Thus, the distribution of oligomers in the lyase degraded SA1 and SA2 samples are very similar, and it would not be a surprise if they were extracted from the same raw material. Besides, both samples contains a significant amount of MG blocks as seen from the ΔG signal. This dimer is a result of the M-lyase specificity towards G-M linkages. The samples contains less G than *L.hyperborea* alginate, revealed by a larger unsaturated mannuronan trimer (ΔM3) peak, as well as a smaller signal for the unresolved peaks late in the chromatograms. Long G blocks, which is a typical feature of brown seaweed alginates, was found in both samples. It should be noted that the PAD detector response is decreasing with chain length [68]. The part of the chromatograms containing oligomers with DP > 30 therefore represents a larger fraction of the sample than the peak areas suggests.

**Normalised antibacterial activity**

The normalised width of the antimicrobial “halo” (*nw_halo_*) of each sample obtained by the agar disk diffusion test against the tested Gram-positive *S. aureus* and methicillin-resistant *Staphylococcus epidermidis* (MRSE) strains and calculated with Equation (1) showed neither statistically significant increase of antibacterial activity by the incorporation of antibacterial GO into the zinc alginates nor by the use of different commercial sodium alginates (see S1 Table).

**S1 Table. Antimicrobial results by the agar disk diffusion test.** Antimicrobial “halo” (*nw_halo_*) of zinc alginate with (A1GO and A2GO) and without (A1 and A2) 1% *w/w* of graphene oxide by the agar disk diffusion test against the tested *S. aureus* and MRSE strains and calculated with Equation (1). The negative results obtained with the control calcium alginate samples (C1 and C2) are also indicated.

| **Sample** | *S. aureus* | MRSE |
| --- | --- | --- |
| **C1** | 0.00±0.00 | 0.00±0.00 |
| **A1** | 0.48±0.03 | 0.49±0.03 |
| **A1GO** | 0.48±0.01 | 0.48±0.03 |
| **C2** | 0.00±0.00 | 0.00±0.00 |
| **A2** | 0.48±0.01 | 0.48±0.04 |
| **A2GO** | 0.48±0.03 | 0.49±0.05 |
